# Temporal patterns in adolescent psychiatric treatment and outcomes: a nationwide register-based cohort follow-up

**DOI:** 10.1007/s00787-024-02554-z

**Published:** 2024-08-14

**Authors:** Tomi Bergström, Kari Valtanen, Jouko Miettunen, Tapio Gauffin, Mia Kurtti

**Affiliations:** 1 Department of Psychology, University of Jyväskylä, Jyväskylä, Finland; 2 Department of Psychiatry, Wellbeing Services County of Lapland, Kemi, Finland; 3 Research Unit of Population Health, University of Oulu, Oulu, Finland; 4 Medical Research Center Oulu, Oulu University Hospital and University of Oulu, Oulu, Finland; 5 Faculty of Medicine, University of Helsinki, Helsinki, Finland; 6 Department of Strategic Services, Wellbeing Services County of Lapland, Rovaniemi, Finland; 7 Faculty of Social Sciences, University of Helsinki, Helsinki, Finland

**Keywords:** Cohort study, Adolescents, Mental health services, Real-world outcome

## Abstract

The rise in mental health problems among adolescents in high-income countries presents a challenge to service systems. For the development of services, there is a need for better insight into temporal psychiatric treatment-trends and outcomes. This study aims to analyze time-trends in both psychiatric treatment patterns and outcomes, utilizing a national sample of all adolescents receiving psychiatric treatment in Finland from 2003 to 2013. For time-trend-analysis, the sample was divided into two cohorts, using the onset year of 2008 as a cutoff. For each case, information on psychiatric treatment was gathered from registers within a five-year follow-up period from the onset of treatment or to death. The association between the inclusion year and outcome variables was studied via weighted generalized linear models. Adolescents in the latter cohort had a greater proportion (*p* < 0.001) of mood and anxiety diagnoses, a lower likelihood of hospitalization, a higher average of outpatient visits, and greater usage of psychotropics (excluding benzodiazepines). Those whose treatment began after 2008 were more likely to be alive (baseline characteristic adjusted Odds Ratio (aOR): 0.7, 95%CI: 0.6–0.8) and still in treatment contact (aOR: 1.4, 95%CI: 1.3–1.4) after four years from the onset. There was no difference in the long-term disability ratio. The results indicate favorable developments towards outpatient care in mental health services for adolescents with a significant decrease in mortality. Approaches to further developing cost-effective, personalized mental health services are discussed.

## Introduction

There is a significant peak in mental health problems during adolescence [1]. The globally estimated prevalence of common mental disorders in adolescents is 25% [2], making them a leading cause of disability among adolescents in high-income countries [3]. Additionally, adolescent psychiatric symptoms significantly predict mental health disorders in adulthood [4] and are associated with an increased risk of suicide, which ranks among the leading causes of death for adolescents [5].

Epidemiological data from previous years reveals consistent trends in the treatment and diagnosis of mental disorders. In the United States, there has been a growing number of adolescents seeking care for internalizing mental health problems such as depression and anxiety, with an increasing proportion receiving specialty outpatient care [6]. Similar observations have been reported in European high-income countries. For example, in Finland from 2000 to 2011, there was an increase in the diagnoses of depression, anxiety disorders, attention deficit disorders, and eating disorders, while the diagnoses of psychosis and conduct disorders decreased, and the average length of hospital stays dropped [7]. After 2011, the proportion of diagnoses of depression, anxiety disorders, and ADHD continued to increase in adolescent psychiatric wards, while diagnosed psychosis and length of hospital stays decreased [8].

Finnish epidemiological data on adolescent mental disorders aligns with the global transformation of mental health care services from institutional settings towards community-oriented care, generally considered more humane than traditional institutional care [9]. In Finland, one example of an initiative aiming to strengthen community-based services and implement evidence-based treatments, especially for young people with mental health difficulties, was the publication of the national plan for mental health work in 2009 [10], accompanied by the national development program for social welfare and healthcare in 2008 [11].

Even though an increase in internalizing mental health problems and usage of mental health services can be interpreted as an indicator of reduced stigma and a greater willingness to seek help at lower thresholds, this may pose challenges for the mental health service system. In Finland, there has been a significant increase in referrals to mental health services, and the waiting lists for psychiatric care have exponentially increased from 2014 to 2020 [12]. Given that rapid response in mental health problems improves treatment outcomes [1], this trend may negatively impact the overall effectiveness of adolescent mental health services.

For development of more effective services, there is a need for more information on nationwide time-trends in psychiatric treatment practices and the longer-term outcomes of adolescent mental health treatment. This study aims to analyze time trends in both the psychiatric treatment patterns and outcomes, utilizing a national sample of all adolescents receiving mental health treatment in Finland from 2003 to 2013. Based on previous literature, we hypothesized that adolescents whose treatment commenced in later inclusion years would demonstrate a higher prevalence of internalizing mental health disorders, a reduced hospitalization rate, and enhanced long-term outcomes. We anticipated that this improvement would manifest in reduced duration of treatment, as well as in decreased disability allowances and mortality ratios.

## Materials and methods

A longitudinal register-based follow-up included all adolescents aged 13–20 who enrolled in psychiatric services in Finland between January 1, 2003, and December 31, 2013, according to the care register for healthcare. Data for each case were gathered from national social and health registers in 2020 and 2021. The data included all available entries up to the end of the year 2018, enabling a fixed continuous follow-up for each case from the onset of adolescent psychiatric treatment until death or the 5-year follow-up, whichever occurred first.

For comparative purpose, cohort was divided into two groups based on the year of onset (prior and after 2009), which aligned with the publication of the national plan for mental health. Note, that the use of the 2009 cut-off year serves mainly as a reference for time-trend analysis, since the publication of national plan doesn’t ensure the immediate adoption of different practices, and there are likely to be multiple time-related factors influencing treatment and outcomes.

### Measures

To form the baseline, treatment pattern, and outcome variables, we utilized data from various registers. Baseline variables were age at the onset of adolescent psychiatric treatment, sex, the usage of child psychiatric services, child protective services prior to the onset of adolescent psychiatric treatment and psychiatric diagnoses within the first year from the onset of adolescent psychiatric treatment. Since in Finland, the eastern and northern parts of the country had higher prevalence and incidence rates of severe mental disorders [13], this categorization was also included as one of the baseline variables.

The treatment pattern variables were number of hospital admissions, days spent in hospital, number of outpatient visits, psychotropic purchases, cumulative medication exposure (sum of DDDs of the psychotropics divided by the follow-up days minus hospital days lacking information on medication usage), disability pensions, and disability expenses (in euros) during the five-year follow-up.

The five-year outcome variables were treatment contact at the end of the five-year follow-up (Yes: if there were one or more visits to specialized or primary mental healthcare units, and/or psychiatric hospital days, between days 1460–1825 from onset (i.e. last follow-up year)), psychotropics at the end of the follow-up (Yes: if there psychiatric medication purchases and/or psychiatric medication used in hospital, between days 1460–1825 from onset) disability pension at the end of the follow-up (Yes: if there were one or more payments of full or partial disability allowances, or cash rehabilitation benefits, granted due to psychiatric disorders, between days 1460–1825 from onset), and death during the follow-up.

### Statistical analysis

Descriptive statistics were used to summarize the baseline characteristics of the sample. Population proportions were used to evaluate temporal changes in five-year usage of services separately for each inclusion year. Outliers were detected and trimmed via Tukey’s fence. Group differences were compared via Chi-square and T-tests.

Stabilized inverse probability of treatment weighting (SIPTW) [14] was employed to adjust for demographical and clinical baseline characteristics between the cohorts of 2003–2008 and 2009–2013, aiming to assess whether time-related changes in psychiatric treatment patterns and outcomes were independent of patient-related characteristics. Propensity scores for SIPTW were calculated for each case through multivariable logistic regression. To analyze service usage at the end of the follow-up, the follow-up time was also adjusted for to address losses due to deaths.

The association between the inclusion cohort and treatment and outcome variables was examined using SIPTW-weighted generalized linear models with a logit link function. Statistical significance was determined to be *p* < 0.05. Adjusted odds ratios (aOR) with 95% confidence intervals (95%CI) were used to assess the direction and strength of the association. Post-hoc comparisons were conducted by including only adolescents with poor outcome (those who were receiving mental health disability allowances at the end of the follow-up or who have died).

Because the limited availability of information regarding outpatient treatment at the primary level prior to 2011 could potentially bias some of the outcome estimation, additional sensitivity analysis was conducted including only adolescents whose treatment began after 2005.

All analyses were conducted via IBM SPSS Statistics 29 for Windows.

## Results

### Sample characteristics

**Fig. 1:**
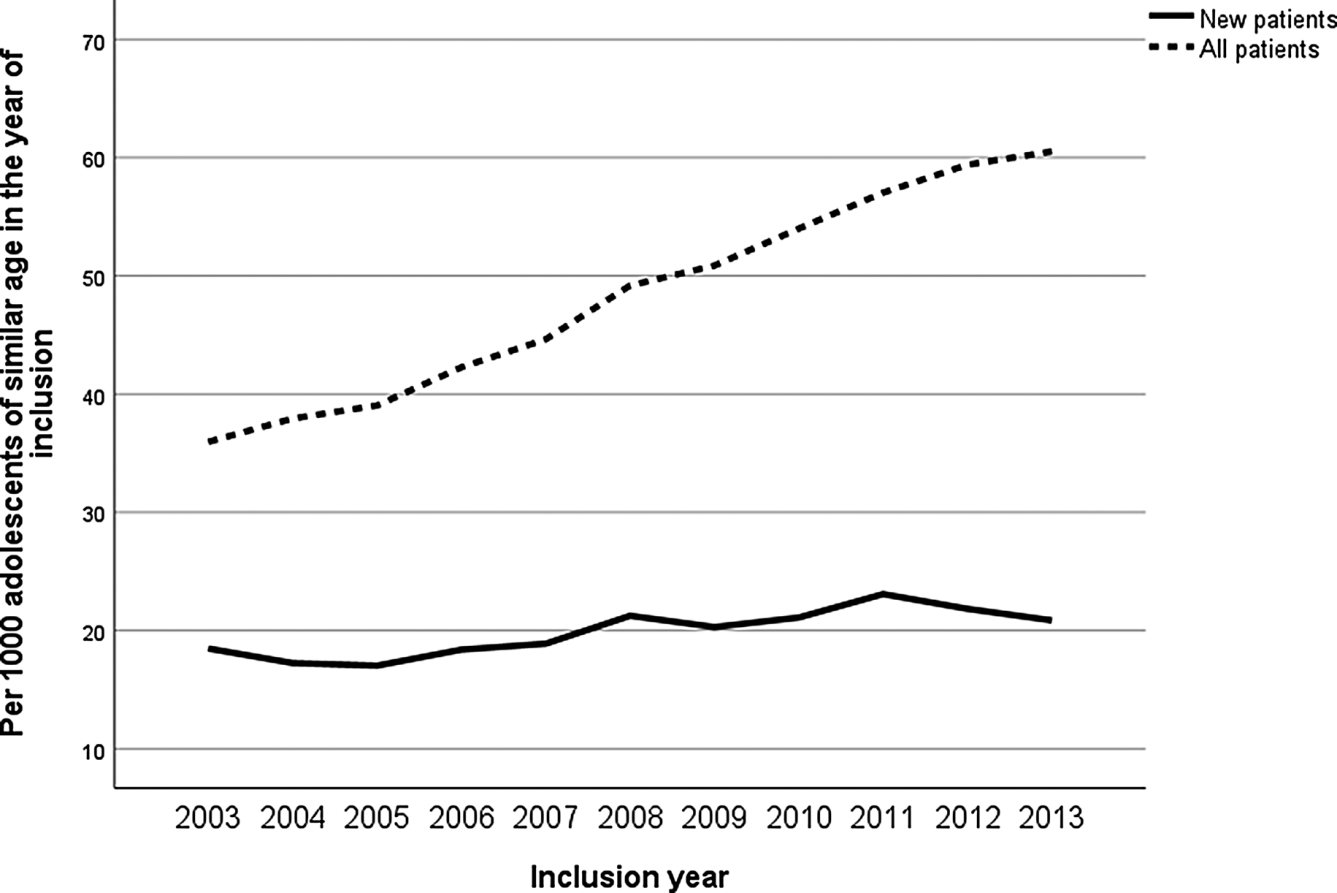
New and total number of adolescent psychiatric patients per inclusion year

**Fig. 2:**
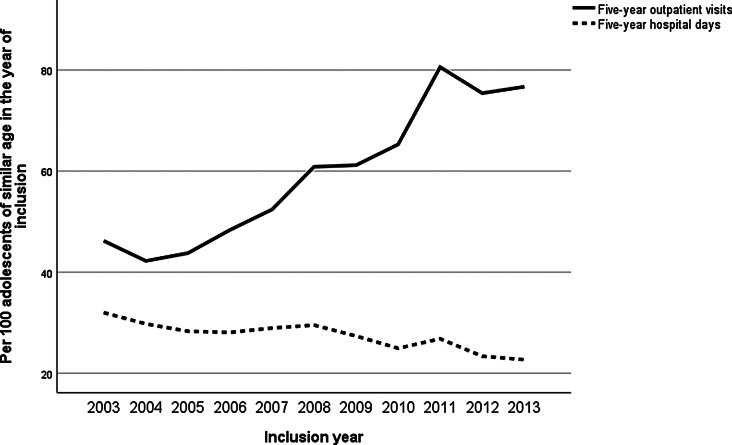
Five-year hospital- and outpatient ratio per inclusion year

**Fig. 3:**
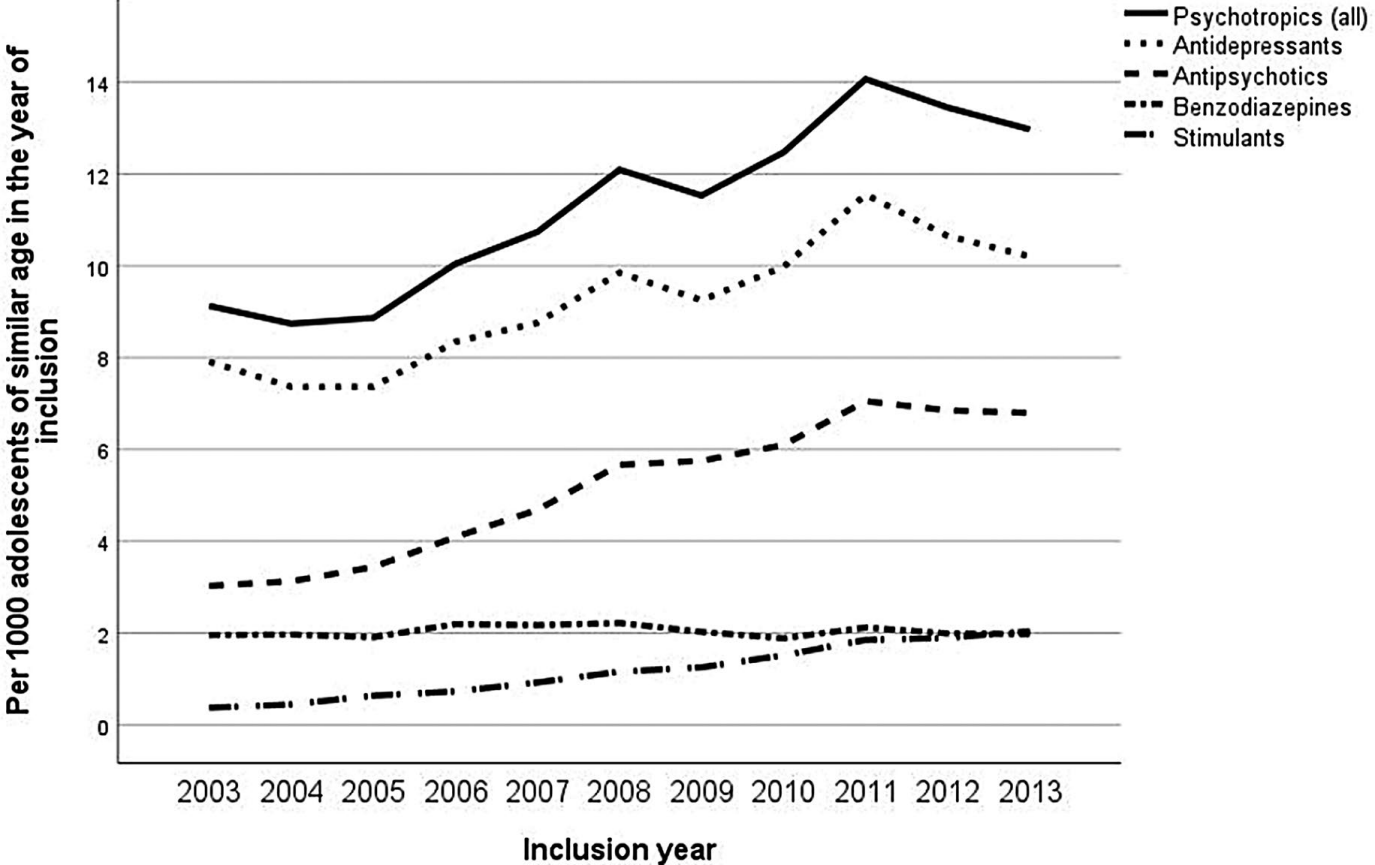
Five-year psychotropic usage per inclusion year

Throughout the inclusion years, the annual prevalence of adolescent psychiatric patients, including also those whose treatment began before 2003, increased more sharply than the incidence of new adolescent patients (Fig. 1). This suggests that individuals entering treatment in later years tended to remain in services for a longer duration. The overall frequency of outpatient visits in five-year follow-up per adolescent of similar age was higher in adolescents who came into the treatment in latter inclusion years, while the length of hospital stays decreased (Fig. 2). This trend was accompanied by an overall increase in the utilization of all psychotropic, except benzodiazepines, which remained relatively stable throughout the inclusion years (Fig. 3).

Average onset age of adolescent psychiatric treatment was 15.6 years (standard deviation, SD: 2.3), 66,586 (59%) of patients were female and 20,787 (19%) had received psychiatric treatment at childhood. Most common baseline primary diagnosis was mood disorder (F3) (24%), followed by anxiety disorder (F4) (21%) and behavioural disorders (F9) (16%). 47,990 (43%) adolescents were still receiving mental health treatment and/or disability allowances at the end of the five-year follow-up, and 759 (1%) had died during the follow-up.

### Time-trend analysis

Adolescents who enrolled to services after 2008 were younger at the beginning of their treatment, and they were more likely to have received child psychiatric and protective services prior to starting adolescent treatment. They had a higher likelihood of being diagnosed with a mental health disorder within the first year of treatment, with a greater proportion of diagnoses related to mood disorders, anxiety disorders, neurodevelopmental disorders, and behavioural disorders (Table 1). The proportion of diagnoses related to psychoses and substance-related disorders was lower. Adolescents in the latter cohort had a lower likelihood of being hospitalized, and for those who were hospitalized, their average duration of stay was shorter. Instead, there was a higher average number of outpatient visits and greater usage of psychotropic medications, particularly antidepressants, antipsychotics and psychostimulants.

**Table 1:** The demographic, clinical, and outcome characteristics of the sample with and without weighting

	Non-weighted sample			Baseline-characteristics weighted sample		
Cohort	2003–2008 *n* = 57,808	2009–2013 *n* = 54,700	p	2003–2008 *n* = 57,785	2009–2013 *n* = 54,717	p
Baseline						
Age (mean (sd))	15.7 (2.5)	15.6 (2.3)	< 0.001	15.6 (2.2)	15.6 (2.2)	0.9
Sex, Women	34,263 (59%)	32,323 (59%)	0.5	34,218 (59%)	32,390 (59%)	0.9
East/North^a^	16,678(29%)	14,419(26%)	< 0.001	16,020 (28%)	15,172 (28%)	0.9
Child psychiatric services	9455 (16%)	11,332 (21%)	< 0.001	10,599 (18%)	10,062 (18%)	0.8
Prior child protective services	6306 (11%)	7034 (13%)	< 0.001	6855 (12%)	6492 (12%)	0.9
Baseline diagnosis^b^						
F0	50 (0.1%)	47 (0.1%)	0.9	53 (0.1%)	49 (0.1%)	0.9
F1	1206 (2.1%)	1045 (1.9%)	0.03	1259 (2%)	1097 (2%)	0.9
F2	1747 (3%)	1527 (2.8%)	0.02	1700 (3%)	1605 (3%)	0.9
F3	13,156 (23%)	14,008 (26%)	< 0.001	13,935 (24%)	13,191 (24%)	0.9
F4	11,121 (19%)	12,670 (23%)	< 0.001	12,189 (21%)	11,544 (21%)	0.9
F5	2841 (5%)	3171 (6%)	< 0.001	3088 (5%)	2924 (5%)	0.9
F6	513 (1%)	533 (1%)	0.2	552 (1%)	519 (1%)	0.9
F7	157 (0.3%)	198 (0.4%)	0.1	173 (0.3%)	185 (0.3%)	0.9
F8	1371 (2%)	2093 (4%)	< 0.001	1752 (3%)	1675 (3%)	0.8
F9	8530 (15%)	9951 (18%)	< 0.001	9468 (16%)	8972 (16%)	0.9
Any F-diagnosis	34,850 (60%)	36,301 (66%)	< 0.001	36,501 (63%)	34,565 (63%)	0.9
Treatment in follow-up						
Hospitalized	14,955 (26%)	12,405 (23%)	< 0.001	15,489 (27%)	12,055 (22%)	< 0.001
Hospital days (mean (SD))	61 (65)	52 (59)	< 0.001	61 (65)	52 (59)	< 0.001
Outpatient visits (mean (SD))	27 (31)	31 (35)	< 0.001	27 (31)	32 (34)	< 0.001
Psychotropics	30,980 (54%)	32,898 (60%)	< 0.001	31,428 (54%)	32,411 (60%)	< 0.001
DDD per day (mean (SD)) ^c^	0.36 (0.42)	0.38 (0.42)	< 0.001	0.37 (0.42)	0.37 (0.42)	0.2
Antidepressants	25,772 (45%)	26,345 (48%)	< 0.001	26,091 (45%)	26,050 (48%)	< 0.001
DDD per day (mean (SD)) ^c^	0.31 (0.33)	0.34 (0.34)	< 0.001	0.32 (0.33)	0.33 (0.34)	< 0.001
Antipsychotics	12,501 (22%)	16,595 (30%)	< 0.001	12,871 (22%)	16,148 (30%)	< 0.001
DDD per day (mean (SD)) ^c^	0.12 (0.13)	0.09 (0.10)	< 0.001	0.11 (0.12)	0.10 (0.10)	< 0.001
Benzodiazepines	6451 (11%)	5104 (9%)	< 0.001	6442 (11%)	5060 (9%)	< 0.001
DDD per day (mean (SD)) ^c^	0.06 (0.07)	0.04 (0.06)	< 0.001	0.06 (0.07)	0.04 (0.06)	< 0.001
Psychostimulants	2221 (4%)	4355 (8%)	< 0.001	2347 (4%)	4127 (8%)	< 0.001
DDD per day (mean (SD)) ^c^	0.31 (0.37)	0.35 (0.40)	< 0.001	0.32 (0.37)	0.34 (0.40)	0.02
Disability in follow-up						
Disability allowance	11,332 (20%)	8194 (15%)	< 0.001	11,520 (20%)	8084 (15%)	< 0.001
Euro (mean (SD))	61 K (56 K)	52 K (37 K)	< 0.001	61 K (56 K)	51 K (37 K)	< 0.001
Mental health sickness allowance	8194 (14%)	8146 (15%)	< 0.001	8287 (14%)	8117 (15%)	< 0.05
Euro (mean (SD))	3 K (2 K)	4 K (3 K)	< 0.001	3 K (3 K)	4 K (3 K)	< 0.001
Income support	26,948 (47%)	26,249 (48%)	< 0.001	27,036 (47%)	26,224 (48%)	< 0.001
Euro (mean (SD))	7 K (7 K)	8 K (8 K)	< 0.001	7 K (7 K)	8 K (8 K)	< 0.001
Five-year outcome						
Treatment contact^d^	15,032 (26%)	18,576 (34%)	< 0.001	15,345 (27%)	18,283 (33%)	< 0.001
Psychotropic^e^	16,441 (28%)	16,962 (31%)	< 0.001	16,700 (29%)	16,667 (31%)	< 0.001
Disability^f^	6639 (12%)	6434 (12%)	0.1	6736 (12%)	6355 (12%)	0.8
Length (mean (SD))^g^	774 (684)	927 (692)	< 0.001	787 (684)	913 (694)	< 0.001
Death	455 (0.8%)	304 (0.6%)	< 0.001	453 (0.8%)	306 (0.6%)	< 0.001
Suicide	202 (0.3%)	119 (0.2%)	< 0.001	204 (0.4%)	120 (0.2%)	< 0.001
Time to death in days (mean (SD))	931 (543)	815 (570)	< 0.01	934 (543)	803 (570)	< 0.01

*Abbreviations* SD, standard deviation; DDD, defined daily dose

^a^Yes, if treatment was initiated in eastern or northern parts of Finland, where the prevalence rate of severe mental disorders is higher

^b^Yes, if there is one or more entries with the diagnosis in question during the first year of follow-up

^c^Including only adolescents with one or more purchases of the medication in question

^d^Yes, if there were one or more visits to specialized or primary mental healthcare units, and/or psychiatric hospital days during the final follow-up year

^e^Yes, if there were one or more psychiatric medication purchases during the final follow-up year

^f^Yes, if there were one or more mental health disability allowances or sickness leave during the final follow-up year

^g^Average treatment duration in days (calculated as the difference between the last entry and the first entry)

In the earlier cohort, a higher proportion of adolescents received benzodiazepines. Among those who received antipsychotics in the earlier cohort, the cumulative exposure was higher, indicating higher dosages. In earlier cohort more adolescents received mental health disability allowances at some point of the follow-up, while the proportion of adolescents receiving income support and short-term mental health sickness allowances was lower.

After adjusting for baseline characteristics, the initiation of treatment between 2009 and 2013 was found to be significantly associated (*p* < 0.001) with a reduced likelihood of hospitalization during the follow-up (aOR: 0.77, 95%CI: 0.75–0.79) and an increased likelihood of receiving psychotropic treatment (aOR: 1.2, 95%CI: 1.1–1.2) as compared to earlier cohort. At the end of the follow-up, adolescents in the latter cohort were also more likely to still be receiving psychotropics (aOR: 1.1, 95%CI: 1.05–1.1) and/or other psychiatric treatments (aOR: 1.4, 95%CI: 1.3–1.4; sensitivity analysis including only adolescents whose treatment initiation occurred after the year 2005: aOR: 1.2, 95%CI: 1.1–1.3), and they were more likely to be alive at the end of the follow-up (aOR: 0.7, 95%CI: 0.6–0.8). No statistically significant differences were observed between the 2003–2008 and 2009–2013 cohorts in disability ratio at the end of the follow-up (aOR: 0.9, 95%CI: 0.9–1.03).

### Post hoc analysis of poor outcomes

The first post hoc analysis focused on adolescents who were still receiving disability allowances at the end of the follow-up. In the latter cohort, a higher proportion of adolescents who were disabled at the end of the follow-up, were initially treated for mood and anxiety disorders, while fewer of them received treatment for psychotic disorders (Table 2). Similarly, a lower number of adolescents in the latter cohort were hospitalized, and the overall utilization ratio of psychotropics was higher. In the latter cohort, disability expenses were lower, while expenses related to mental health sickness and income support were higher. Furthermore, more adolescents in this cohort were still receiving psychiatric treatment at the end of the follow-up.

In the second post hoc analysis, which focused on adolescents who died during the follow-up, there were no statistically significant differences in diagnostic distribution or other baseline characteristics between the two cohorts. However, among the adolescents in the earlier cohort who died, there was a higher likelihood of hospitalization during the follow-up, fewer outpatient visits, and a greater likelihood of receiving antidepressants and/or benzodiazepines prior to their death.

**Table 2:** Post-hoc comparisons including only adolescents with disability allowances at the end of the follow-up and those who had died

	Disability allowances at the end of the follow-up			Death		
Cohort	2003–2008 *n* = 6639	2009–2013 *n* = 6434	p	2003–2008 *n* = 455	2009–2013 *n* = 304	p
Baseline						
Age (mean (sd))	15.6 (2.3)	15.6 (2.3)	0.8	17 (2.3)	17 (2.3)	0.4
Gender, Women	2823 (43%)	2666 (41%)	0.2	155 (34%)	114 (38%)	0.3
East/North^a^	1981 (30%)	1768 (28%)	0.003	151 (33%)	97 (32%)	0.7
Child psychiatric services	1256 (19%)	1529 (24%)	< 0.001	50 (11%)	47 (18%)	0.1
Prior child protective services	1027 (16%)	1124 (18%)	< 0.05	62 (14%)	49 (16%)	0.3
Baseline diagnosis^b^						
F0	15 (0.2%)	15 (0.2%)	0.9	< 5 (< 1%)	< 5 (< 1%)	0.8
F1	152 (2%)	129 (2%)	0.3	63 (14%)	35 (12%)	0.3
F2	678 (10%)	519 (8%)	< 0.001	40 (9%)	25 (8%)	0.8
F3	1790 (27%)	1907 (30%)	< 0.001	121 (27%)	89 (29%)	0.4
F4	1318 (20%)	1500 (23%)	< 0.001	99 (22%)	71 (23%)	0.6
F5	274 (4%)	308 (5%)	0.07	13 (3%)	14 (5%)	0.2
F6	80 (1%)	88 (1%)	0.4	5 (1%)	5 (2%)	0.5
F7	83 (1%)	119 (1%)	0.005	< 5 (< 1%)	< 5 (< 1%)	0.3
F8	230 (4%)	325 (5%)	< 0.001	< 5 (< 1%)	< 5 (< 1%)	0.6
F9	990 (15%)	1126 (18%)	< 0.001	38 (11%)	38 (13%)	0.5
Any F-diagnosis	4522 (68%)	4582 (71%)	< 0.001	313 (69%)	207 (68%)	0.8
Treatment						
Hospitalized	3461 (52%)	2970 (46%)	< 0.001	215 (47%)	119 (39%)	< 0.05
Hospital days (mean (SD))	98 (74)	83 (72)	< 0.001	56 (64)	51 (61)	0.6
Outpatient visits (mean (SD))	47 (40)	57 (41)	< 0.001	19 (26)	23 (29)	< 0.05
Psychotropics	5110 (77%)	5243 (82%)	< 0.001	309 (68%)	205 (67%)	0.9
DDD per day (mean (SD)) ^c^	0.60 (0.50)	0.60 (0.50)	0.1	0.63 (0.58)	0.64 (0.54)	0.8
Antidepressants	4331 (65%)	4358 (68%)	< 0.05	249 (55%)	144 (47%)	< 0.05
DDD per day (mean (SD)) ^c^	0.44 (0.40)	0.45 (0.40)	0.1	0.71 (0.54)	0.73 (0.58)	0.7
Antipsychotics	3495 (53%)	3800 (59%)	< 0.001	169 (37%)	123 (41%)	0.4
DDD per day (mean (SD)) ^c^	0.17 (0.10)	0.14 (0.10)	< 0.001	0.15 (0.14)	0.15 (0.14)	0.9
Benzodiazepines	1758 (27%)	1458 (23%)	< 0.001	148 (33%)	78 (26%)	< 0.05
DDD per day (mean (SD)) ^c^	0.08 (0.08)	0.05 (0.07)	< 0.001	0.12 (0.08)	0.12 (0.08)	0.8
Psychostimulants DDD per day (mean (SD)) ^c^	303 (5%) 0.35 (0.40)	527 (8%) 0.37 (0.42)	< 0.001 < 0.001	15 (3%) 0.51 (0.60)	13 (4%) 0.51 (0.36)	0.5 0.5
Disability						
Disability allowance	5414 (82%)	4781 (74%)	< 0.001	79 (17%)	60 (20%)	0.4
Euro (mean (SD))	83 K (61 K)	56 K (39 K)	< 0.001	1 K (1 K)	3 K (2 K)	< 0.001
Mental health sickness allowance	3923 (59%)	3969 (62%)	< 0.05	105 (23%)	58 (19%)	0.2
Euro (mean (SD))	4 K (3 K)	5 K (3 K)	< 0.001	2 K (2 K)	4 K (3 K)	< 0.001
Income support	3798 (57%)	3806 (59%)	< 0.05	256 (56%)	162 (53%)	0.4
Euro (mean (SD))	7 K (7 K)	8 K (8 K)	< 0.001	8 K (8 K)	8 K (8 K)	0.8
Five-year outcome						
Treatment contact^d^	4029 (61%)	4404 (68%)	< 0.001	NA	NA	
Psychotropic^e^	4157 (63%)	4229 (66%)	< 0.001	NA	NA	

*Abbreviations* SD, standard deviation; DDD, defined daily dose

^a^Yes, if treatment was initiated in eastern or northern parts of Finland, where the prevalence rate of severe mental disorders is higher

^b^Yes, if there is one or more entries with the diagnosis in question during the first year of follow-up

^c^Including only adolescents with one or more purchases of the medication in question

^d^Yes, if there were one or more visits to specialized or primary mental healthcare units, and/or psychiatric hospital days during the final follow-up year

^e^Yes, if there were one or more psychiatric medication purchases during the final follow-up year

## Discussion

This nationwide register-based cohort follow-up aimed to investigate the psychiatric treatment patterns and treatment outcomes over a five-year period for adolescents who utilized psychiatric services in Finland between 2003 and 2013. Align with previous research [6–9], we observed a notable shift in treatment patterns, particularly towards outpatient care, which seemed to occur at least partially independently of patient characteristics. Furthermore, there was a significant increase in the proportion of adolescents receiving psychotropic medications, particularly antipsychotics and stimulants.

The cumulative medication exposure to antipsychotics was lower, suggesting that this increase was primarily attributed to the off-label usage of antipsychotics. This aligns with previous studies indicating that antipsychotics are being utilized more frequently for the treatment of other problems instead of psychosis [15–17]. While there was a slight decrease in the proportion of adolescents receiving benzodiazepines, the change was modest, and overall, we observed a significant increase in the usage of psychotropic medications. This may indicate a more systematic provision of psychotropics in alignment with current treatment guidelines. For example, it is observed that attention deficit hyperactivity disorders are more frequently diagnosed in recent years, reflecting changes in administrative and clinical practices and likely explaining the increasing rate of stimulant use [18]. In addition to a potentially different threshold for medication, it is possible that the greater overall utilization of psychotropics could also be driven by limited availability of other treatments and services, including nationwide reduction of hospital beds.

The five-year mortality and suicide ratio was significantly lower in adolescents who sought treatment after 2008. The usage of psychotropic medications, particularly antidepressants, has been suggested as a potential explanation for the decline in mortality among individuals with mental health problems [19], and our analysis revealed also a linear increase in the utilization of psychotropics alongside a decrease in mortality ratio. However, in post-hoc analysis adolescents who sought treatment before 2009 and subsequently died were actually more likely to have received antidepressants and benzodiazepines prior to their death, with no other discernible differences in medication treatment patterns. This aligns with recent observations [20, 21], which indicate that the observed declining trend, particularly in suicide rates, is more likely influenced by other time-related factors rather than being a direct consequence of the increased utilization of psychotropic treatments. It is also noteworthy that mortality did not appear to be in a linear relationship with the decrease in hospital care. Although conclusions about causality cannot be drawn from this study, this result may suggest that psychiatric services can be safely developed with a focus on outpatient care without an increase in mortality-ratio.

Adolescents who sought treatment after 2008 were more likely to still be receiving treatment after the five-year follow-up period. Furthermore, there was a significant increase in the annual prevalence of adolescent mental health service users during the inclusion years of the study, indicating that adolescents who sought treatment in later years spent a longer time in services compared to those in previous years. There were no statistically significant differences in the ratio of disability allowances at the end of the follow-up. These findings are particularly noteworthy considering the indications that a higher proportion of adolescents sought psychiatric care, and the proportion of severe psychiatric disorders, such as psychoses and substance abuse disorders, was slightly lower in the latter cohort. Moreover, these results persisted even after adjusting for observable baseline characteristics.

While the observational nature of this study prevents us from drawing causal explanations, there are several potential explanations for this finding. Firstly, the higher usage rates of psychotropic medications, which are often recommended for long-term use, may contribute to the longer treatment periods. Then again, longer usage of services may also reflect systematic efforts to help individuals who are experiencing mental health difficulties, following from the reform of services. It is suggested that the stigma surrounding mental health problems has diminished, leading to better adherence of psychiatric treatment and more individuals seeking help from mental health services [22]. Social network members, including professionals from schools and other services, may also guide adolescents with lower thresholds to psychiatric services.

While increasing awareness and willingness to receive mental health help are positive signs, they also pose a risk of medicalizing mental and social phenomena, leading to an overreliance on medical responses to human life problems. To minimize this, more holistic treatment strategies are suggested [23, 24]. Examples of such strategies, which have preliminary shown reduced cumulative service usage and psychotropic treatments alongside decreased long-term disability ratios, incorporating more contextual and social network-focused approaches [23–27]. Future studies should explore whether these approaches can be more broadly implemented to cost-effectively enhance treatment outcomes for adolescents with mental health difficulties.

### Strength and limitations

Finnish registers are considered as a reliable source of information [28], allowing for the non-selective inclusion of all individuals receiving adolescent psychiatric treatment in Finland. However, registers were not originally designed for research purposes, potentially introducing inaccuracies. Limitations included the lack of information on outpatient primary healthcare prior to 2011 and psychotherapy conducted in the private healthcare sector. Sensitivity analysis focusing on patients with follow-up after 2010 suggested that the missing information on primary-level outpatient care did not bias the main findings. The absence of information on private psychotherapy was also unlikely to impact the main conclusions, given the significant increase in this practice after the 2010s [29].

Since registers were the sole source of information, standardized measures were lacking to estimate the primary outcome. To compensate, strict outcome measures such as survival and non-usage of any services or mental health support at the end of the fixed five-year follow-up were used as proxies for symptomatic recovery, considering that in Finland the healthcare and social services are guaranteed to entire population based on national social insurance. In other words, it is unlikely that there would not be any registered entries of treatments or support in the Finnish system over the long term if the individual’s symptomatology remained disabling. However, this measure does not directly capture symptoms or more existential outcomes, such as subjective experiences of well-being. Disability allowances at follow-up may also be influenced by varying thresholds for receiving such support, which are affected more by policy and financial constraints than individual condition.

Detailed information on symptom severity at onset and time-related factors influencing for example the content of treatment and diagnostic procedures was lacking. Although our approach accounted for evolving and comorbid baseline diagnostic distributions, the potential heterogeneity of diagnostic practices and the possibility of providing treatment without a formal diagnosis necessitate further analysis of different diagnostic combinations and trends during follow-up. Note also that the higher incidence of adolescent patients in the latter cohort suggests an increase in adolescents with less severe symptoms seeking services, which could have particularly impacted outcomes. Due to the limited availability of initial clinical information, the weighting does not fully address this potential confounder.

Due to the aforementioned limitations, the results should be considered as describing rough national trends and changes in adolescent psychiatric treatments, and their associations with long-term outcomes. Note also that the primary objective of this study was not to assess the outcomes of the national mental health plan. Instead, the selection of the 2009 cut-off year was chosen mainly as a historical reference to enable comparison before and after identifiable shifts in mental health regulations and adolescent services. The findings suggest success in this regard, as no extreme weights were observed, and there were noticeable differences in treatment practices regardless of patient characteristics. However, numerous residual factors likely associate with both service intake and treatments, apart from the publication of the national mental health plan. Therefore, a linear causal relationship between the publication of the national plan and treatment practices and outcomes cannot be established.

## Conclusion

In a nationwide register-based follow-up study, encompassing all adolescents in psychiatric care in Finland in 2003–2013, noteworthy temporal changes were observed in psychiatric treatment practices. These changes included an increased emphasis on outpatient treatment, accompanied by a significant reduction in mortality. There was also a notable rise in the utilization of psychotropics and longer treatment durations.

While extended treatment duration can be beneficial in many cases and may partially explain some of the favourable changes observed in time-trend analysis, it also presents challenges, including the risk of overtreatment and increased service caseload. The increased caseload from prolonged service utilization may also partly account for the challenges faced by Finnish mental health services. Given the constraints of limited public resources and a shortage of professionals, it is essential to allocate resources effectively for psychiatric services and to develop cost-effective treatment strategies that safely enhance long-term outcomes.

## Data Availability

The Health and Social Data Permit Authority Findata supervise the secondary use of all Finnish health and social care data including all of the data used in this study. Data can be accessed based on justified purposes. For more information and data permit applications see findata.fi.
